# Functional analysis of the *GmESR1* gene associated with soybean regeneration

**DOI:** 10.1371/journal.pone.0175656

**Published:** 2017-04-12

**Authors:** Chao Zhang, Xiaodong Wu, Binbin Zhang, Qingshan Chen, Ming Liu, Dawei Xin, Zhaoming Qi, Sinan Li, Yanlong Ma, Lingshuang Wang, Yangmei Jin, Wenbin Li, Xiaoxia Wu, An-yu Su

**Affiliations:** 1 Soybean Research Institute, Key Laboratory of Soybean Biology of Chinese Education Ministry, Northeast Agricultural University, Harbin, Heilongjiang province, People’s Republic of China; 2 College of Resources and Environment, Northeast Agricultural University. Harbin, Heilongjiang province, People’s Republic of China; College of Agricultural Sciences, UNITED STATES

## Abstract

Plant regeneration can occur via *in vitro* tissue culture through somatic embryogenesis or *de novo* shoot organogenesis. Transformation of soybean (*Glycine max*) is difficult, hence optimization of the transformation system for soybean regeneration is required. This study investigated *ENHANCER OF SHOOT REGENERATION 1* (*GmESR1*), a soybean transcription factor that targets regeneration-associated genes. Sequence analysis showed that *GmESR1* contained a conserved 57 amino acid APETALA 2 (AP2)/ETHYLENE RESPONSE FACTOR (ERF) DNA-binding domain. The relative expression level of *GmESR1* was highest in young embryos, flowers and stems in the soybean cultivar ‘Dongnong 50’. To examine the function of *GmESR1*, transgenic *Arabidopsis* (*Arabidopsis thaliana*) and soybean plants overexpressing *GmESR1* were generated. In *Arabidopsis*, overexpression of *GmESR1* resulted in accelerated seed germination, and seedling shoot and root elongation. In soybean overexpression of *GmESR1* also led to faster seed germination, and shoot and root elongation. GmESR1 specifically bound to the GCC-box. The results provide a foundation for the establishment of an efficient and stable transformation system for soybean.

## Introduction

Plant regeneration is a clonal propagation process *in vitro*, which may involve a variety of processes, such as exogenous plant hormone signaling response, division of quiescent cells, and formation of a meristem or organ primordia [1]. Overexpression of cyclin-related genes in *Arabidopsis thaliana* could be an important link between cell proliferation in shoot apical meristems and organogenesis [2]. Shaul *et al*. (1996) [3] suggested that the expression of cyclin-dependent kinase genes was highly correlated with acquisition of the ability for cell proliferation. In *Arabidopsis*, the regeneration-associated *CLAVATA* (*CLV*) genes may regulate stem cell fate, such as limiting the size of the stem cell population, and the function of *CLV3* is dependent on *WUSCHEL* (*WUS*) activity in the embryonic shoot meristem [4–6]. A recent study indicates that changes in molecular patterning scales are associated with organ size in apical stem cell niches in plants [7]. Genes that promote cell proliferation or cell volume increase in soybean have not been studied previously.

Plant transformation is achieved by transferring a DNA fragment to the genome of a plant cell, regenerating a shoot from the transgenic cell, and then generating a root system to produce a genetically modified plant [8, 9]. *Rhizobium radiobacter* (*Agrobacterium tumefaciens*) mediated methods or particle bombardment are the preferred DNA transfer techniques [10]. To achieve high frequencies of plant regeneration, Cheng *et al*. (1980) [11] used aseptic cotyledon nodes as explants to induce differentiation of multiple shoots clumps. The genetic transformation of soybean (*Glycine max*) has long been of global interest, with successful transformation dependent on the efficient combination of receptor system and transformation method. The poor reproducibility of soybean regeneration represents a bottleneck for transformation of this important crop. Previous reports on soybean regeneration systems focused mainly on aspects such as genetic constitution, hormone types, culture conditions and explant types. Few studies have investigated the molecular basis of regeneration, especially the underlying mechanisms and the genes involved in this process. Detection of the expression of genes associated with soybean regeneration might help to address this problem.

Many *de novo* organogenesis systems are potentially useful as model experimental systems to illustrate the complexity of plant cell differentiation. The molecular mechanisms activated by the auxin to cytokinin ratio, however, are not well characterized. Efficient shoot regeneration involves two consecutive incubation steps: the exophyte is first incubated on an auxin-rich callus induction medium, and then on a cytokinin-rich shoot induction medium. The auxin rich callus induction medium initiates the formation of organogenic callus. More explants could improve the conversion efficiency, thus increasing the rate of emergence. Many regeneration-associated genes are related to the cytokinin signaling pathway, of which *ENHANCER OF SHOOT REGENERATION 1* (*AtESR1*) is one gene. *AtESR1* plays an important role in the regeneration network [12–15].

APETALA 2/ETHYLENE RESPONSE FACTOR (AP2/ERF) transcription factors (TFs) are involved in various biological functions, including plant and flower development, fruit and seed maturation, pathogen defense, and responses to damage, high salinity, and drought [16]. The AP2/ERF family belongs to a large group of TFs present in all plant species [17]. In *Arabidopsis*, the AP2/ERF TF family is divided into five subfamilies: AP2, Related to ABI3/VP1 (RAV), Dehydration Responsive Element Binding (DREB), ERF, and others [16]. These TFs are characterized by a conserved DNA-binding domain, the AP2/ERF domain, which was originally identified in *Arabidopsis* and is composed of 57–66 amino acids [18–20]. The ERF subfamily is the largest subgroup in the AP2/ERF family with members containing one or two AP2/ERF domains with specific DNA-binding motifs [21–23]. The three-dimensional structure of the AP2/ERF domain protein showed that the region contains three β fold, which is differences from located in the second β fold in the first 14 (alanine) and 19 (aspartic) amino acid residues, determines the specific binding of such TFs to different *cis*-acting elements [24]. Gutterson *et al*. (2004) [25] suggested that ten distinct subfamilies accommodate the structural difference in B subgroup. The ERF VIII-b group genes usually function in the early stages of shoot regeneration [22, 26]. The ERF subfamily of TFs participates in biological stress responses through binding to the GCC-box (AGCCGCC) [27]. The role of ERF TFs in the regulation of shoot regeneration is complex; both their biological function, and the ERF-mediated signal transduction pathway, are not well characterized.

The *AtESR1* gene (also known as *DORNRÖSCHEN*; *DRN*) was identified by screening an *Arabidopsis* cDNA library. The ESR1 protein contains a domain that shows sequence homology to the AP2/ERF domain [20, 28, 29]. *ESR1* appears to regulate shoot differentiation, with overexpression of *ESR1* greatly enhancing the efficiency of shoot regeneration in *Arabidopsis* tissue culture [14]. *ESR1* encodes a TF belonging to the ERF family. The region between the AP2/ERF domain and the ESR motif in *ESR1* is indicated to be essential for enhancement of shoot regeneration [30]. *ESR1* acts as a transcriptional activator [31, 32]. ESR1 binds to the GCC-box *in vitro* [33]. The GCC-box is an ethylene-responsive element located in the promoter region of many pathogenesis-related genes [34, 35]. Using yeast two-hybrid screening, ESR1 has been shown to interact with PHAVOLUTA (PHV), while coimmunoprecipitation and bimolecular fluorescence complementation have shown that ESR1 interacts with BES INTERACTING MYC-LIKE PROTEIN 1 (BIM1), a basic helix-loop-helix (bHLH) protein. BIM1 and PHV also physically interact [36, 37]. In *Arabidopsis*, shoot-related auxin-transport is conducted by ESR1 and ESR2, two partially redundant AP2 TFs that interact during shoot development with PINOID (PID) and PIN-FORMED 1 (PIN1), respectively [38].

An orthologue of *AtESR1* was previously isolated from maize. In the maize shoot apical meristem, similar to *AtESR1*, *ZmESR1* transcriptional activity is associated with the anlage of new lateral organs [39]. In the present study we isolated *GmESR1* (GenBank accession no. JN590243.1, NCBI protein no. AFO52509.2), an AP2/ERF TF containing an ESR motif, from the soybean cultivar ‘Dongnong 50’. In this study we examined one of the two gene copies present in the soybean genome. We present expression patterns of the full-length GmESR1 protein and binding to the GCC-box element and show that the *GmESR1* transcript abundance varies in different organs. *GmESR1* showed organ-specific expression in soybean. Overexpression of *GmESR1* in transgenic soybean and *Arabidopsis* plants was also investigated. Overexpression of *GmESR1* promoted germination and elongation in soybean and *Arabidopsis*.

## Materials and methods

### Plant materials and cultivation condition

For plant transformation, seeds of soybean (*Glycine max* (L.) Merr.) cultivar ‘Dongnong 50’, which shows a high frequency of regeneration, were obtained from the Key Laboratory of Soybean Biology in the Chinese Ministry of Education, Harbin. Seedlings were grown in a growth chamber maintained at 26°C/18°C (day/night) under a 16 h photoperiod and light intensity of 350 μmol·m^−2^·s^−1^. Transgenic T_1_ soybean seeds were sown under the same conditions. Fifteen days after planting, seedlings at the first-node stage (soybean growth phase V1) [40] were used for phenotype analysis and expression analysis using quantitative real-time PCR (qRT-PCR).

*Arabidopsis thaliana* ecotype Columbia (Col-0) was used as the wild type (WT). For transgenic *Arabidopsis*, the T_3_ generation was used. Seeds of the mutant *Arabidopsis atesr1* T-DNA insertion line (Salk_089567) were obtained from The Arabidopsis Information Resource.

### Isolation of *GmESR1*

To identify *GmESR1* and homologs in other plant species, the Phytozome 11.0 (https://phytozome.jgi.doe.gov/pz/portal.html) database was searched using the *AtESR1* gene sequence, extracted from the National Center for Biotechnology Information (NCBI) website, as the query sequence. Total RNA was reverse-transcribed into single-stranded cDNA using the ReverTra Ace® qPCR RT Kit (TOYOBO, Japan). Using this cDNA as a template, *GmESR1* gene-specific primers (*GmESR1-*F/R) were used to amplify *GmESR1*. PCR reaction conditions were as follows: 94°C for 4.5 min, then 35 cycles at 94°C for 30 s, 58°C for 30 s, and 72°C for 1.5 min, with final extension at 72°C for 10 min. The PCR products were inserted into the pMDTM19-T vector and ligated together (Takara, Japan). The ligation products were then transformed into *Escherichia coli* DH5α cells (TIANGEN, China) and sequenced (BioMed, China). The sequences were aligned with the *GmESR1* sequence using BLAST (http://www.ncbi.nlm.nih.gov/BLAST). The isoelectric point and the molecular weight of the GmESR1 protein were analyzed using the Swiss Institute of Bioinformatics Compute pI/Mw tool (http://web.expasy.org/compute_pi/). Nucleotide and amino acid sequences were compared using the sequence alignment software DNAMAN 6.0 (http://www.lynnon.com/). To predict the GmESR1 protein structure and discover potential domains, the InterPro online portal was used (https://www.ebi.ac.uk/interpro/). Analysis of homologous protein sequence similarity was performed using the algorithm blastp (protein–protein BLAST) (http://www.ncbi.nlm.nih.gov/blast). Phylogenetic analysis of a multiple sequence alignment of the amino acid sequences of GmESR1 and heterologous AP2/ERF members was performed using MEGA 5.2 software (http://www.megasoftware.net). The three-dimensional structure of GmESR1 was predicted using the Phyre 2 online portal (http://www.sbg.bio.ic.ac.uk/phyre2). The RasMol software 2.7.2.1.1 (http://www.OpenRasMol.org/Copyright.html) was used to generate a graphical representation of the protein structure.

### Real-time RT-PCR analysis of *GmESR1* expression

The expression of *GmESR1* was examined with qRT-PCR using SYBR^®^ Premix Ex Taq™ II Kit (Tli RNaseH Plus, Takara) according to the manufacturers’ instructions (Takara), on an ABI 7500 Real-Time PCR Detection System (ABI, USA). Total RNA was extracted from the pod, root, stem, leaf, flower and immature embryo of soybean ‘Dongnong 50’ plants using TRIzol^®^ Reagent according to the manufacturers’ protocol (Invitrogen, China). Genomic DNA was removed, and reverse transcription carried out, using the PrimeScript™ RT Reagent Kit with gDNA Eraser (Takara). Approximately 1 μg of total RNA was used for each reaction. To remove genomic DNA, samples were incubated at 42°C for 2 min. For reverse transcription, each reaction used 10 μl of the reaction solution from the first step in a total volume of 20 μl. Samples were incubated at 37°C for 15 min followed by heat shock at 85°C for 5 s. A standard two-step PCR amplification protocol of 95°C for 30 s, followed by 45 cycles at 95°C for 5 s and 60°C for 40 s, was used. Gene-specific primers (*GmESR1*-qF/R) for *GmESR1*, the soybean internal control gene *GmACTIN4* (GenBank accession no. AF049106) and the *Arabidopsis* internal control gene *AtACTIN8* (*A*. *thaliana* 18S rRNA gene GenBank accession no. X16077) were used. *GmACTIN4* and *AtACTIN8* were used as reference genes. The 2^−ΔΔCt^ method was used to determine the relative level of *GmESR1* expression in different tissues. Three technical replicates were performed for each real-time RT-PCR experiment.

### Expression and purification of recombinant GmESR1 protein

The full-length coding region of *GmESRl* was amplified using gene-specific primers (*GmESR1-*1F/R). The PCR products were digested with *Bam*HI and *Hin*dIII and were inserted into the pET-29b vector (EMD Millipore, USA). The recombinant vector pET29b-*GmESR1* was transformed into BL21 (DE3) competent *E*. *coli* cells, which were then grown in Luria broth (LB) with 50 mg·mL^−1^ kanamycin at 37°C to an absorbance of 0.7 at 600 nm. The *E*. *coli* liquid medium was induced with 0.5 mM isopropyl β-D-1-thiogalactopyranoside (IPTG). After 4 h induction, the cells were isolated via centrifugation at 5000 ×*g* for 12 min at room temperature. To purify the recombinant protein, bacteria were resuspended in 15 ml of 1× binding buffer and kept on the ice for 25 min. This was followed by cycles of ultrasonification for 20 s and pause for 20 s until the sample was no longer sticky. The sample was then centrifuged at 2000 ×*g* for 8 min at room temperature before being recycled and loaded onto a His-bind Resin column (Novagen, BRD). The pure GmESR1 fusion protein was analyzed by sodium dodecyl sulfate-polyacrylamide gel electrophoresis (SDS-PAGE) and quantified based on the pET System manufacturer’s protocol (Novagen, BRD).

### Electrophoretic mobility shift assay

In soybean and Arabidopsis, members of the ERF family contain a conserved DNA-binding domain (AP2/ERF domain) [41]. A digoxigenin–ddUTP-labeled double-stranded oligonucleotide GCC-box probe has previously been combined with the DNA-binding activity of soybean Ethylene Response Factor 5 (GmERF5) [42]. The sequence of the GCC-box probe and the mutated GCC-box probe are shown in S1 Table. The electrophoretic mobility shift assay (EMSA) was performed as described by Kass *et al*. (2000) [43].

### Identification of transgenic *atesr1* plants

Plants homozygous for the T-DNA insert were identified via PCR using a gene-specific primer pair and a T-DNA-specific primer. The left genomic primer (LP), right genomic primer (RP) and the left T-DNA border primer (LB) for *atesr1* are shown in S1 Table. After confirmation of the homozygous T-DNA insertion, reduction in relative gene expression level was confirmed using qRT-PCR with a gene-specific primer.

### Construction of *GmESR1* overexpression vector

To overexpress *GmESR1* under the control of the *Cauliflower mosaic virus* (CaMV) 35S promoter, the pEarleyGate 101 vector, containing the *bar*^*r*^ gene, was used via the Gateway cloning system. The full-length open reading frame sequence of *GmESR1* was used by designing flanking primers for the BP reaction. The reaction mixture was: 1 μl pGWC, 2 μl buffer, 1 μl T4 ligase, 4 μl gene fragment and water to 10 μl, with the ligation carried out at 16°C. Next, the fragment with adapters at each end was cloned into the entry vector pGWC, which contains chloramphenicol resistance. The entry clone pGWC-*GmESR1* was used to perform the LR reaction. The extracted pEarleyGate 101 plasmid, 1 μl of each entry clone, 1 μl LR enzyme, and water to make up the volume to 5 μl was incubated at 25°C to facilitate the recombination reaction and clone the desired fragment into the pEarleyGate 101 destination vector. The *R*. *radiobacter* strain LBA4404 was transformed with the overexpression vector using the freeze–thaw method as described by Dang *et al*. (2007) [44].

### *Arabidopsis* transformation and phenotype analysis

Using the method described by Clough *et al*.(1998) [45], *Arabidopsis* was transformed with the overexpression vector. *Arabidopsis* seeds were vernalized in the dark at 4°C. The seeds were sterilized in 10% sodium hypochlorite, vortexed for 10 min and washed six times using distilled sterile water. The seeds were sown on Murashige and Skoog (MS) solid medium and, after 3–4 leaves had developed, the seedlings were transplanted into 1:1 sterilized soil and vermiculite mixture. Transformation via infiltration was carried out during flowering. *Rhizobium radiobacter* cells from a single colony were suspended in 15 ml Yeast Extract Peptone liquid culture medium containing selection antibiotics and incubated at 28°C, with shaking at 185 rpm, until the absorbance at 600 nm was approximately 0.5. When the absorbance at 600 nm reached 1.6–2.0 the mixture was centrifuged for 15 min at 5000 ×*g*, and the supernatant was discarded. The flowering *Arabidopsis* plants were inverted and immersed in the *Rhizobium* liquid for 30 s. Plants were covered with plastic film and placed in a thermostatic chamber without light for 24 h, then placed upright and left to grow with ambient illumination.

The *atesr1* mutant lines, transgenic *GmESR1* overexpression lines, and WT *Arabidopsis* plants were grown in the same growth chamber, maintained at 22°C with a 16 h/8 h (light/dark) cycle and light intensity of 350 μmol·m^−2^·s^−1^. After 2.5 d, the rates of germination and elongation in the *GmESR1* overexpression lines, *atesr1* mutant lines, and WT plants were compared and analyzed statistically. After flowering, the height of *Arabidopsis* were compared.

### Soybean transformation and phenotype analysis

*Rhizobium*-mediated stable soybean transformation was performed using cotyledonary nodes of soybean ‘Dongnong 50’ as explants. Following culturing in the dark, shoot regenerative proliferation, shoot elongation induction, root differentiation multiplication, and plantlet regeneration, the regenerated plants were transferred to pots and grown in the greenhouse [46]. Five T_1_ *GmESR1*-overexpressing soybean plants and control lines were grown on soybean seed germination medium. After 5 days, each cotyledon separation and 7–8 wounds were induced in the growing point, to the co-culture medium and dark culture for 3 days, transfer into the bud induction medium; co-culture for 14 d at 25°C under the same conditions. Then the same size buds of *GmESR1*-overexpressing and control group was isolated and used for observion by electron microscopy.

## Results

### Isolation and molecular characterization of cDNA clone encoding *GmESR1*

**Fig 1:**
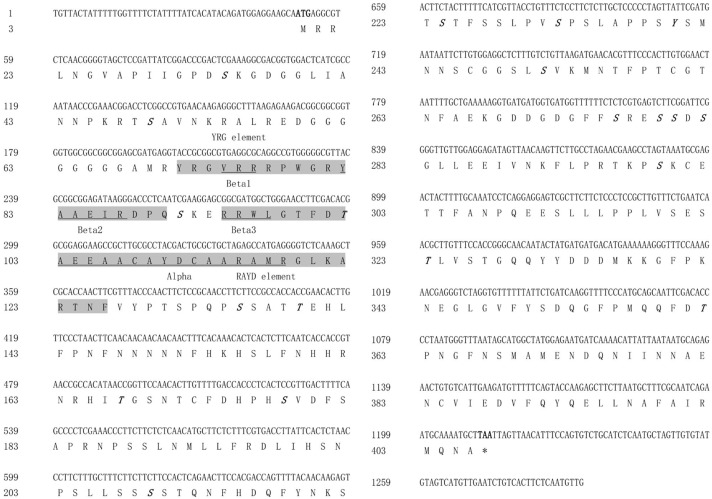
Nucleotide and amino acid sequences of *GmESR1*. Putative phosphorylation sites are marked in bold italics. The YRG element and RAYD element are highlighted by shading. The α-helix and β-sheets are underlined. Amino acid and base pair numbers are shown on the left.

The full-length *GmESR1* cDNA sequence of 1,292 bp, containing an open reading frame of 1,164 bp and encoding a protein of 387 amino acids (GenBank accession no. JN590243.1), was obtained from soybean ‘Dongnong 50’ (Fig 1). The GmESR1 protein was predicted to have a molecular mass of 42.8324 kDa and an isoelectric point at pH 6.80. The nucleotide sequence showed a 5′ untranslated region (UTR) of 49 nucleotides and a 3′ UTR of 79 nucleotides. The NetPhos 2.0 online server (http://www.cbs.dtu.dk/services/NetPhos-2.0/) predicted that GmESR1 contained 14 serine residues (Ser 15, 30, 72, 116, 159, 190, 205, 213, 232, 257, 260, 261, 263 and 280), five threonine (Thr 83, 120, 148, 304 and 343), and one tyrosine (Tyr 221), which were potential phosphorylation sites (Fig 1).

**Fig 2:**
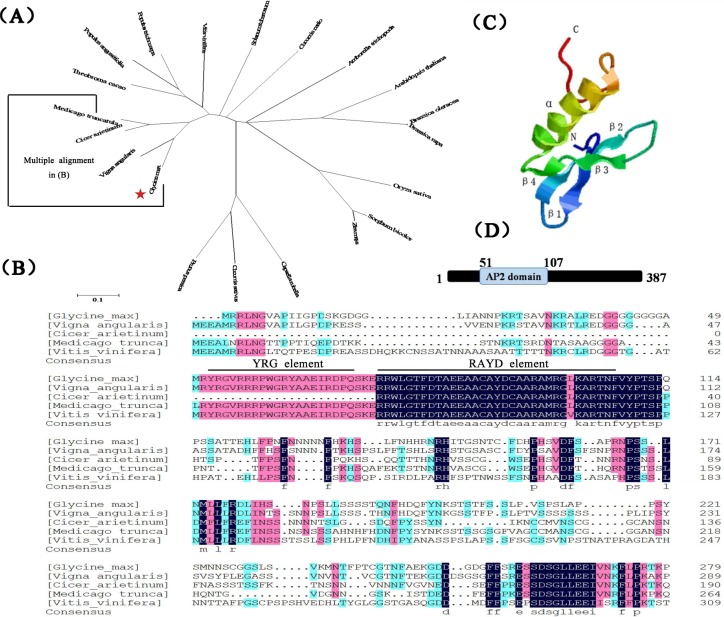
Characterization of *GmESR1*. (A) Phylogenetic analysis of GmESR1 with 20 other ESR1 proteins The GenBank accession numbers are as follows: *Glycine max* [JN590243], *Vigna angularis* [KOM39777], *Cicer arietinum* [XP_004489775], *Medicago truncatula* [XP_003613106], *Vitis vinifera* [XP_002271778], *Theobroma cacao* [XP_007044810], *Populus angustifolia* [AGA18050], *Populus trichocarpa* [XP_002314597], *Solanum tuberosum* [XP_006357626], *Cucumis melo* [XM_008446150], *Amborella trichopoda* [XM_006852288], *Arabidopsis thaliana* [NM_101169], *Brassica oleracea* [XM_013746383], *Brassica rapa* [XM_009119902], *Oryza sativa* [Oryza sativa Japonica Group] [NP_001047305], *Sorghum bicolor* [XM_002452333], *Zea mays* [NM_001153873], *Capsella rubella* [EOA21975], *Cucumis sativus* [XP_004152327] and *Prunus persica* [EMJ26264]. (B) Alignment of amino acid sequences of GmESR1 and the four most similar ESR1 proteins. The YRG element and RAYD element are indicated by horizontal lines above the sequence. Amino acid numbers are indicated on the right. (C) The predicted three-dimensional structure of GmESR1. (D) The conserved domain of the GmESR1 protein. The predicted GmESR1 protein contains a conserved domain at amino acids 51–107 that belongs to the AP2 superfamily.

Analysis of the homolog of *GmESR1* in the soybean genome, based on data obtained from the Phytozome database, indicated that the two genes were clustered in two linkage groups, one each on chromosomes Gm 01 and Gm 02, with one and no introns, respectively. Cladistic analysis of GmESR1 against other ESR1s, representing a range of species including crops, fruits, and vegetables, grouped GmESR1 with members of the plant ESR1 family (Fig 2A). The amino acid sequence of GmESR1 showed 68% and 51% similarity to ESR1 proteins from adzuki bean (*Vigna angularis*; KOM39777) and chickpea (*Cicer arietinum*; XP_004489775), respectively (Fig 2B). The predicted three-dimensional structure of *GmESR1*, based on data from Phyre 2, indicated that the protein contained a long C-terminal α-helix (α) wrapped in a three-stranded anti-parallel β-sheet (from β1 to β3) (Fig 2C) and that the AP2/ERF domain was divided into conserved segments (YRG and RAYD) (Fig 2B) [47]. The predicted structure of *GmESR1* included a conserved region of 57 amino acid residues (residues 51–107) representing the predicted AP2/ERF DNA-binding domain (Fig 2D). Given that residue 14 of the domain was an alanine and residue 19 an aspartic acid, the gene was classified as a member of the ERF subfamily of AP2/ERF TFs. These two amino acids are crucial for specific binding of ERF TFs to the GCC-box in promoter regions and to activate transcription of target genes [16].

### Analysis of *GmESR1* expression in various organs

**Fig 3:**
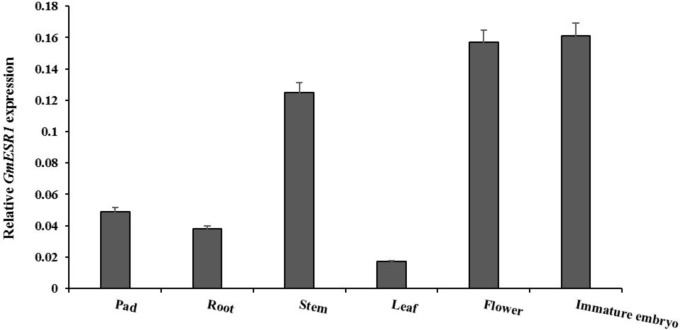
Expression patterns of *GmESR1* in different organs of the soybean cultivar ‘Dongnong 50’. Transcript abundances were normalized against the reference gene *GmActin4*. Bars and error bars represent the mean ± standard error of three experiments with independent RNA extractions.

To investigate the potential role of *GmESR1*, its expression profiles were analyzed in major organs of the soybean plant using qRT-PCR. *GmESR1* was expressed in all organs analyzed, with the highest relative expression levels observed in young embryos, flower and stem tissue, and considerably lower relative expression levels observed in the pod, leaf and root (Fig 3). *GmESR1* may, therefore, play a role in stem elongation, flower morphogenesis, and embryogenesis.

### Purification of the recombinant GmESR1 protein

**Fig 4:**
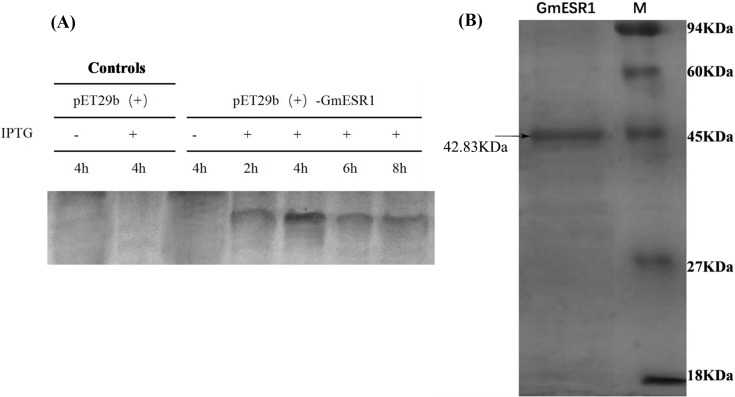
Analysis of the purified recombinant GmESR1 protein. (A) The recombinant GmESR1 protein, induced with 0.5 mM IPTG at 37°C for 2, 4, 6, and 8 h in *E*. *coli* BL21 competent cells. (B) SDS-PAGE analysis of the purified recombinant GmESR1 protein using the His-Bind kit.

Expression of the recombinant GmESR1 protein was markedly enhanced after 2–8 h induction with 0.5 mM IPTG at 37°C, attaining the maximum expression level after 4 h, although the recombinant GmESR1 protein was not detected in the control groups (Fig 4A). The molecular weight of the purified GmESR1 protein was approximately 45 kDa as estimated with SDS-PAGE, consistent with the calculated molecular mass of 42.8324 kDa (Fig 4B).

### GmESR1 specifically binds to the GCC-box element *in vitro*

**Fig 5:**
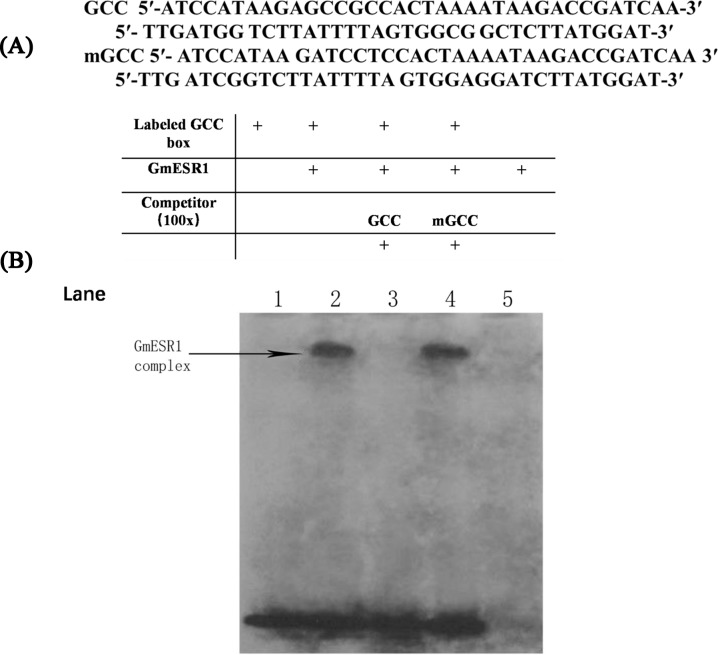
Sequence-specific binding of GmESR1 to the GCC-box element. (A) Nucleotide sequences of the GCC-box and mGCC-box probes. (B) Electrophoretic mobility shift assay (EMSA) showed sequence-specific binding to the GCC-box of the recombinant GmESR1 protein. Lane 1, EMSA performed with only the free GCC probes; lane 2, labeled GCC probe and GmESR1 protein; lane 3, titration with a cold GCC sequence as a competitor; lane 4, titration with a cold mGCC-box sequence as a competitor; lane 5, labeled mGCC probe and GmESR1 protein.

To confirm binding of GmESR1 to the GCC-box regulatory element *in vitro*, His-tagged GmESR1 was purified and used in an EMSA alongside a digoxigenin-ddUTP-labeled double-stranded oligonucleotide GCC-box probe. The GCC-box and mGCC-box sequences are shown in Fig 5A. GmESR1 specifically recognized and bound to the GCC-box, but not to the mGCC-box (Fig 5B). When the ratio of unlabeled to labeled GCC probe was 100:1, the labeled probe was not bound, but when 100-fold unlabeled mGCC probe was used as the competitor, the labeled probe was again bound, confirming the specificity of mobility shift (Fig 5B).

### Analysis of *Arabidopsis atesr1* mutants and overexpression phenotypes in *Arabidopsis* plants

**Fig 6:**
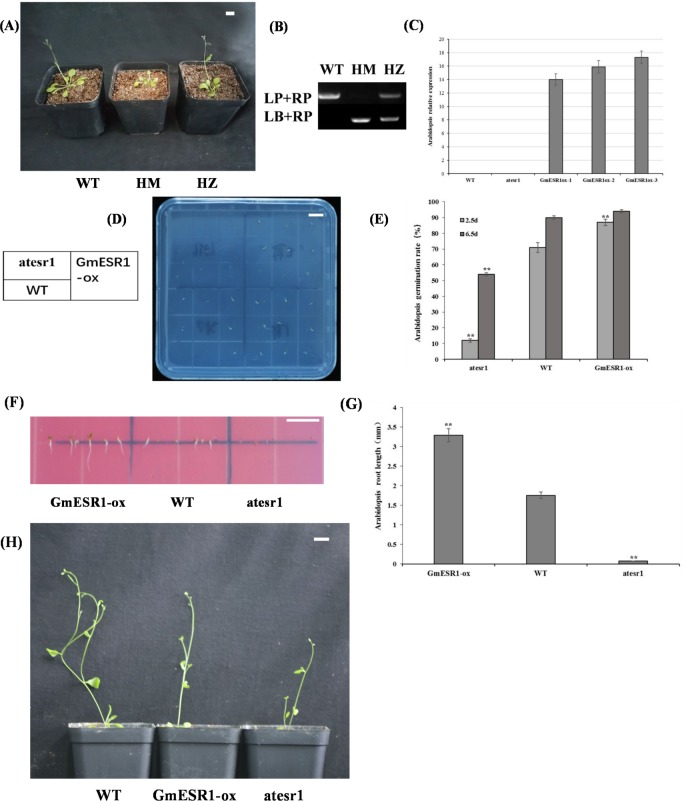
Identification and analysis of mutant *atesr1* and *GmESR1*-ox *Arabidopsis* plants. (A) Phenotypes of homozygous *atesr1* mutant (HM), heterozygous *atesr1* mutant (HZ) and wild-type plant (WT). Scale bar = 1.0 cm. (B) PCR results for the genotyping assay to identify *atesr1* mutant plants. (C) Identification of transgenic *GmESR1*-ox *Arabidopsis* plants using qRT-PCR. Transcript abundances were normalized against the reference gene *AtActin8*. (D) Germination of mutant *atesr1*, WT, and *GmESR1*-ox *Arabidopsis* seeds. Two independent *GmESR1*-ox lines are included. (E) Germination of *atesr1*, WT, and *GmESR1*-ox seeds after 2.5 and 6.5 d. (F) Comparison of *GmESR1*-ox, WT, and *atesr1* elongation rates on MS medium 2.5 d after planting. (G) Root length in *GmESR1*-ox, WT, and mutant *atesr1* plants 2.5 d after planting. (H) Phenotypes of WT, *GmESR1*-ox, and *atesr1* plants 30 d after transplanting. The experiment was performed on three biological replicates with their respective three technical replicates and statistically analyzed using Student’s *t*-test (*P<0.05, **P<0.01). Error bars represent the standard error of the mean.

Using the “double primer” genomic PCR method described by T-DNA Primer Design (http://signal.salk.edu/tdnaprimers.2.html), homozygous T-DNA insertion mutants were screened for the presence of the transgene, resulting in identification of plants homozygous and heterozygous for *atesr1* (Fig 6A and 6B). All WT and transgenic plants were grown under the same conditions. The relative expression level of *GmESR1* was examined in WT, mutant *atesr1*, and three independent *GmESR1*-ox lines. In the three *GmESR1*-ox lines, the transcript abundance of *GmESR1* was high, whereas no transcripts were detected in the WT or *atesr1 Arabidopsis* plants (Fig 6C). This finding confirmed that these three lines were overexpression of *GmESR1*. The germination rates of mutant *atesr1* and *GmESR1*-ox seeds sown on solid medium were compared with those of WT seeds. After 2.5 d, the homozygous mutant *atesr1* displayed poor germination. Compared with *GmESR1*-ox, the germination rates of WT and *atesr1* seeds were both reduced, though WT seeds showed slightly better germination rates than *atesr1* seeds. All *GmESR1*-ox seeds successfully germinated (Fig 6D). The germination rates of WT, *atesr1* and *GmESR1*-ox seeds are shown in Fig 6E. The germination rate after 2.5 and 6.5 d was higher in *GmESR1*-ox seeds compared with WT seeds, and higher in WT seeds compared with *atesr1* seeds. This finding indicated that overexpression of *GmESR1* promoted and accelerated *Arabidopsis* seed germination, whereas the *atesr1* mutation repressed or delayed germination. The elongation rate of *GmESR1-*ox plants was significantly faster than that of WT plants, whereas *atesr1* mutants failed to germinate (Fig 6F). After 2.5 d *GmESR1*-ox seedlings had longer roots than the WT seedlings, and *atesr1* seeds had not germinated (Fig 6G). These findings indicated that *GmESR1* overexpression promoted germination and root elongation, whereas the *atesr1* mutation delayed germination. At 30d after transplanting, it was observed that *GmESR1* overexpression in transgenic *Arabidopsis* plants resulted in dwarfism (Fig 6H).

### Identification and analysis of transgenic soybean overexpressing *GmESR1*

**Fig 7:**
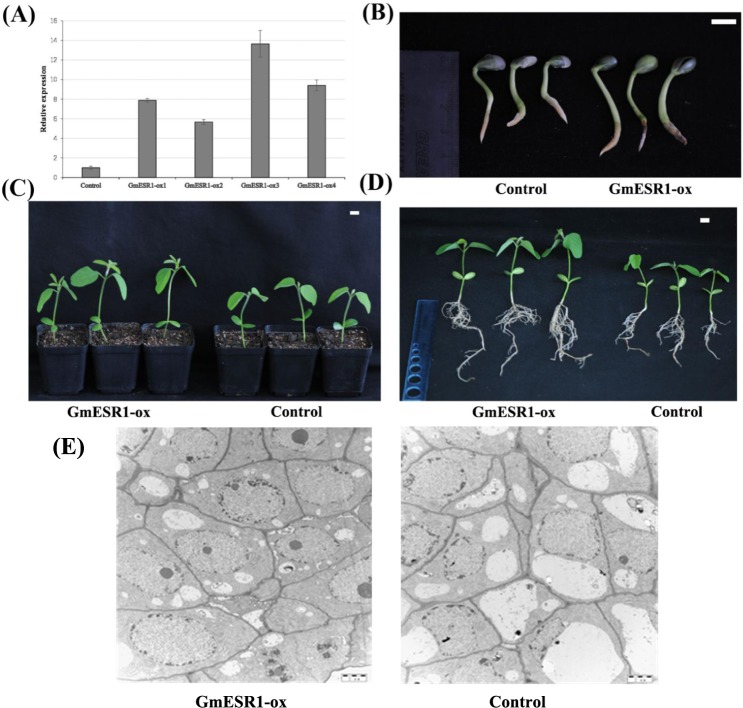
Analysis of *GmESR1* transgenic soybean plants. (A) Relative expression level of *GmESR1* in control group and four independent *GmESR1*-ox lines. Transcript abundance was normalized against the reference gene *GmActin4*. (B) Comparison of elongation rate in *GmESR1*-ox and control soybean plants during germination 5 d after planting. (C) Comparison of shoot elongation rate in *GmESR1*-ox and control soybean seedlings 15 d after planting. (D) Comparison of root elongation in *GmESR1*-ox and control soybean seedlings 15 d after planting. The experiments were performed on three biological replicates with their respective three technical replicates. Error bars represent the standard error of the mean. Scale bars = 1.0 cm. (E) Comparison of the bud cells in *GmESR1*-ox and control soybean plants during the bud induction. Scale bars = 5μm.

The soybean cotyledons were used for stable transformation. T_1_ seeds were sown in soil, and transformants were identified at the V1 developmental stage when the first trifoliate leaf appeared. Transgenic soybean plants overexpressing *GmESR1* were identified via qRT-PCR as those showing higher relative expression of *GmESR1* compared with control soybean plants (Fig 7A). Phenotypic analysis of the transgenic soybean plants showed that *GmESR1* overexpression resulted in faster germination and elongation relative to the control soybean plants, indicating that *GmESR1* promoted seed germination (Fig 7B). After 15 d, at the seedling stage of development, trifoliate leaves had not yet developed in the control soybean but were fully visible in *GmESR1*-ox plants (Fig 7C). At the same time point, the root elongation of *GmESR1*-ox soybean plants was faster and the root length was longer than in control soybean plants (Fig 7D). This finding indicated that *GmESR1* promoted shoot and root elongation in soybean. The experimental results showed that *GmESR1* overexpression resulted in the increasing number of cells relative to the control soybean clustered bud cells in the same size of the field of vision (Fig 7E). It makes the cell division more exuberant.

## Discussion

In this study, we identified *GmESR1* in soybean, a novel member of the ERF family of TFs, that promoted soybean germination, and shoot and root elongation when overexpressed. Nakano *et al*. (2006) [22] investigated phylogenetic relationships, gene structure, and the conserved domain of the ERF gene family in *Arabidopsis* and rice, but only identified a few members of the ERF family in soybean. At 145 and 420 Mb respectively, *Arabidopsis* and rice have small genomes compared with the genome of soybean (1115 Mb) [48]. The structure of the AP2/ERF superfamily are relatively similar in all three species [22]. Presently, 98 genes of known function of the ERF family that contain a complete AP2/ERF domain have been identified in the soybean genome [41]. Only six of these genes have been functionally characterized in soybean [42, 47, 49, 50, 51, 52].

*GmESR1* is the first soybean ERF family TF expressed in response to germination and shoot and root elongation, although little information on the role of *GmESR1* in these processes is available. Although *Arabidopsis AtESR1* genes were first identified in 2001 [14], little is known about their protein functions *in vivo* or *in vitro*. In the present study, *GmESR1* was shown to contain an AP2/ERF domain divided into two conserved segments, the YRG and RAYD elements. The amino acid sequence, protein structure, results of soybean transformation and other biological functions of the GmESR1 protein indicated that *GmESR1* belongs to the ERF subfamily of AP2/ERF TFs. The *GmESR1* sequence analysis indicated that features such as the molecular mass, predicted eukaryotic protein phosphorylation sites, acidic isoelectric point and lack of introns are conserved. *GmESR1* is located on chromosome two and contains no introns. *AtESR1* and *AtESR2* occur as a duplication on chromosome one, with genetic data indicating that they are highly redundant during embryonic patterning [36, 53]. *GmESR1* was analyzed according to publicly available data (http://soybase.org/GlycineBlastPages/) that indicated that 20 genes were clustered into 20 linkage groups.

In embryogenic shoot growth, the cytokinin-induced regenerative genes *AtWUSHEL* (*AtWUS*) triggers TOPLESS (TPL) [54], which weakens auxin signaling by interacting with MONOPTEROS/Auxin Response Factor5 (MP/ARF5) and INDOLE-3-ACETIC ACID INDUCIBLE 12/BODENLOS (IAA12/BDL) [55, 56]. Banno *et al*. (2001) [14] isolated and characterized a novel cDNA of which overexpression promotes ultimate cytokinin-independent shoot regeneration from *Arabidopsis* explants. Given that the cDNA obtained via screening depended on its overexpression as a substitute of cytokinin essential for shoot regeneration, the cDNA might encode elements involved in cytokinin signaling. Overexpression of *AtESR1* in *Arabidopsis* under the control of the estradiol-inducible XVE system also increases shoot regeneration in the presence of cytokinins [14, 57]. The function of *AtESR2* is similar to *AtESR1*, with plants silenced for *AtESR2* displaying weaker regeneration in general, and increased shoot regeneration in the presence of extra cytokinins [58]. The present results indicated that *GmESR1* is responsible for regulation of stem elongation and embryogenesis in soybean and *Arabidopsis*. As plant regeneration is a complex process requiring the interaction of multiple genes, a single gene is insufficient to regulate the entire process and, therefore, additional study of the interactions between *GmESR1* and other regeneration-associated genes is required.

Overexpression of *AtESR1* and *AtESR2* in *Arabidopsis* has previously been shown to upregulate *CUP-SHAPED COTYLEDON 1* (*AtCUC1*) expression, with the upregulation of *AtCUC1* having a positive effect on shoot regeneration [59]. *AtCUC1* overexpression is reported to promote the formation of adventitious buds from callus [60]. *AtESR1* is important in the conversion of the young lateral root primordium into a shoot meristem, whereas *AtESR2* functions in shoot development [61]. *AtCUC1* is involved in the same pathway, and *AtESR2* and *AtCUC2* are functionally redundant in cotyledon development [58]. A previous study of *AtESR1* identified phenotypic changes only during embryonic development, whereas our research analyzed the entire plant growth period. Overexpression of *GmESR1* in transgenic soybean promoted germination and elongation, resulting in faster shoot development than in WT soybean plants, suggesting that *GmESR1* accelerates soybean development and might be a useful tool for regulation of soybean seedling, stem, and root elongation. Overexpression of *GmESR1* in *Arabidopsis* promoted germination and elongation, resulting in faster shoot development than in WT and mutant *atesr1 Arabidopsis* plants, suggesting that overexpression of *GmESR1* accelerates seedling growth stage in *Arabidopsis* and might be a useful tool for regulation of germination, seedling development, and promotion of root elongation. Conversely, *atesr1* delayed germination and the seedling stage in *Arabidopsis*. In mature transgenic *Arabidopsis* plants, overexpression of *GmESR1* resulted in a dwarf phenotype, whereas the mutant *atesr1* showed reduced stem elongation. We have focused on complementation of the *Arabidopsis* mutant, the relative experiment is complicated and tedious, and no data have been obtained as yet.

Here we demonstrated that overexpression of *GmESR1* in soybean and *Arabidopsis* plants improved shoot germination and elongation, and that purified GmESR1 protein binds to the GCC-box, which is present in the promoter region of many genes. These observations are further supported by confirmation of the interaction of the AP2/ERF domain of AtESR1 with class III homeodomain-leucine zipper (HD-ZIP) TFs [36]. The binding specificity of the AtESR1 protein can be changed by the interaction of the AP2/ERF domain of AtESR1 and the C-terminal Per/Arnt/Sim (PAS)-like domain of class III HD-ZIP proteins, thus a prolonged sequence containing the GCC-box can be recognized by a combination of ESR1 and class III HD-ZIP TFs [31]. The AP2-type TFs DRN and DRNL interact with the bHLH protein AtBIM1, which supports a role for AtBIM1 in embryonic patterning [37]. The interactions between the soybean homologs of AtBIM1 and AtPID and GmESR1s are currently under investigation. At present, research into GmESR1 is at an early stage and additional investigations are needed to clarify its involvement in regeneration. Furthermore, other reasons should be sought to explain why such a unique proteinic structure is formed. The *GmESR1* overexpression resulted in more vacuoles relative to the control soybean clustered bud cells in the same size of the field of vision, so can be divide into multiple cells. The *GmESR1* overexpression resulted in larger relative to the control soybean clustered bud cell nucleus. After that, the number of cells increased gradually, which could be showed that soybean plants were growing rapidly at germination and seedling stage. Neighboring cells division increased, and the cells divided repeatedly, and the number of divisions increased linearly [62]. During growth and development, the population of stem cells rapidly proliferates to fill the tissues and organs [63]. The *GmESR1* gene is functionally analogous to animal stem cells, and the ability to regenerate can increase the number of cells and increase the volume of cells.

The work reported here may be used to further elucidate the division between the regulation of defense mechanisms and shoot regeneration by the ERF family. In the present study, seed germination, and shoot and root growth of *GmESR1-*overexpressing transgenic soybean plants were faster than those of non-transgenic soybean plants, suggesting that *GmESR1* may be involved in the regulation of seed germination, and shoot and root elongation.

## Conclusion

We analyzed the function of the soybean *GmESR1* gene. In addition, the relationship between the function of *GmESR1* and seed germination, and shoot and root elongation was investigated. In soybean *GmESR1* overexpression led to faster seed germination, and shoot and root elongation. And by the observation of cell number under the overexpression of *GmESR1*, the result support that *GmESR1* could promote regeneration. These results indicated that *GmESR1* may played an important role in seed germination and elongation of soybean.

## Supporting information

S1 Fig Phylogenetic analysis of GmESR1 and 20 ESR1 proteins from other plant species. (TIF)

S2 Fig PCR analysis of T_1_ transgenic soybean plants using *bar* and *GmESR1* gene-specific primers. (TIF)

S3 Fig Germination of mutant *atesr1*, wild type, and *GmESR1*-ox *Arabidopsis* seeds after 2.5 d. Two independent *GmESR1*-ox lines are included.
(TIF)

S4 Fig Germination of mutant *atesr1*, wild type, and *GmESR1*-ox *Arabidopsis* seeds after 4.5 d. Two independent *GmESR1*-ox lines are included.
(TIF)

S5 Fig Germination of mutant *atesr1*, wild type, and *GmESR1*-ox *Arabidopsis* seeds after 6.5 d. Two independent *GmESR1*-ox lines are included.
(TIF)

S1 Table Oligonucleotide primers used in this study. (DOC)
