# Correction: Functional analysis of the *GmESR1* gene associated with soybean regeneration

**DOI:** 10.1371/journal.pone.0181576

**Published:** 2017-07-12

**Authors:** The PLOS ONE Staff

There are errors in the Funding section. The correct funding information is as follows: This research was suppported through funding from The Funds for Creative Research Groups of Heilongjiang Province of China (JC2016004), Project of outstanding academic leaders in Harbin (2015RQXXJ018), and National Key Research Program of China (2016YFD0100201-21). The funders had no role in study design, data collection and analysis, decision to publish, or preparation of the manuscript. The publisher apologizes for the errors.
