# Supplementary figures and images for: Functional analysis of the GmESR1 gene associated with soybean regeneration
Source: PLoS One. 2017 Apr 12;12(4):e0175656. doi: 10.1371/journal.pone.0175656 (PMC5389854; doi:10.1371/journal.pone.0175656)

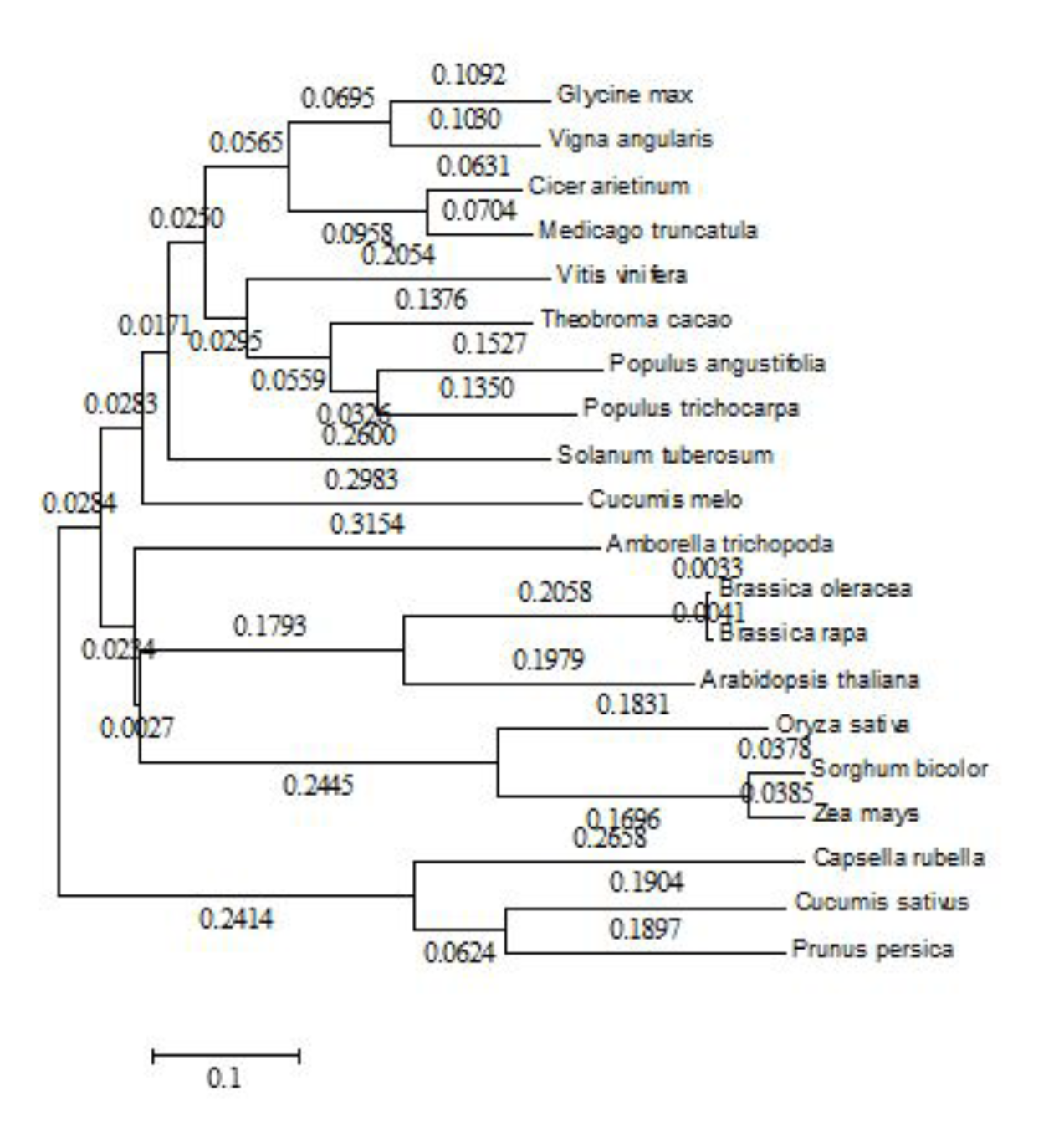

Supplement: S1 Fig — (TIF) [file pone.0175656.s001.tif]

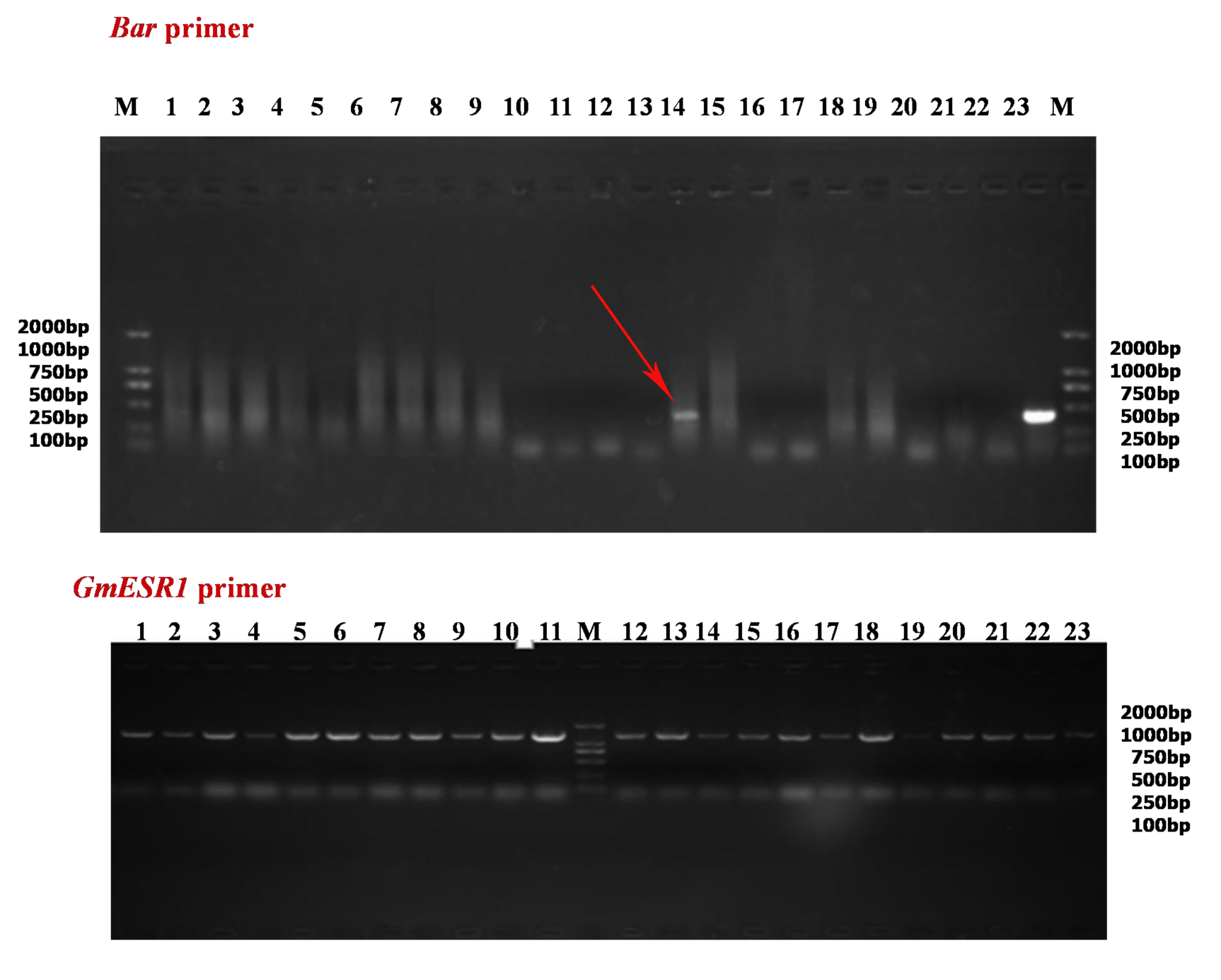

Supplement: S2 Fig — (TIF) [file pone.0175656.s002.tif]

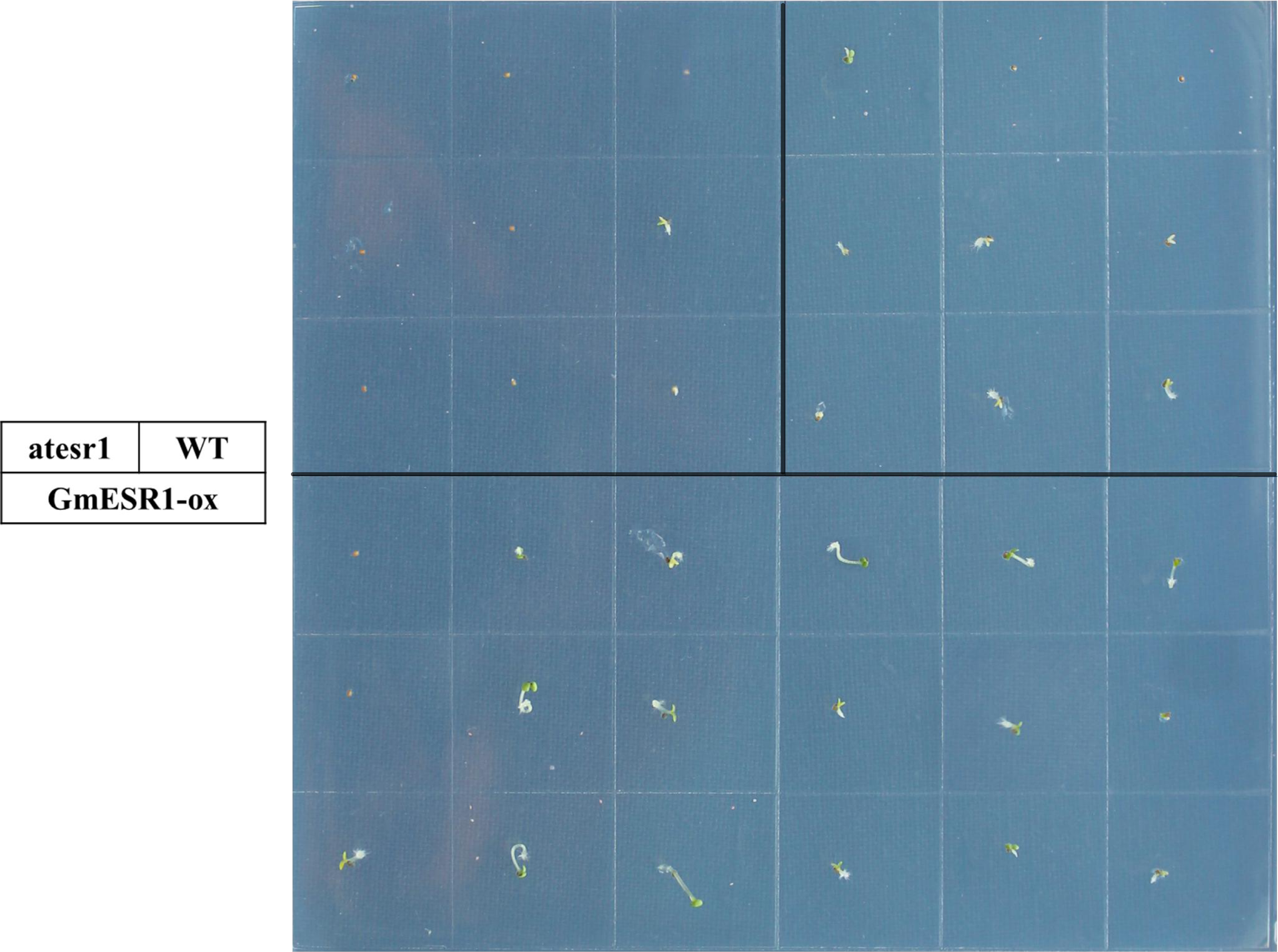

Supplement: S3 Fig — Two independent GmESR1-ox lines are included. (TIF) [file pone.0175656.s003.tif]

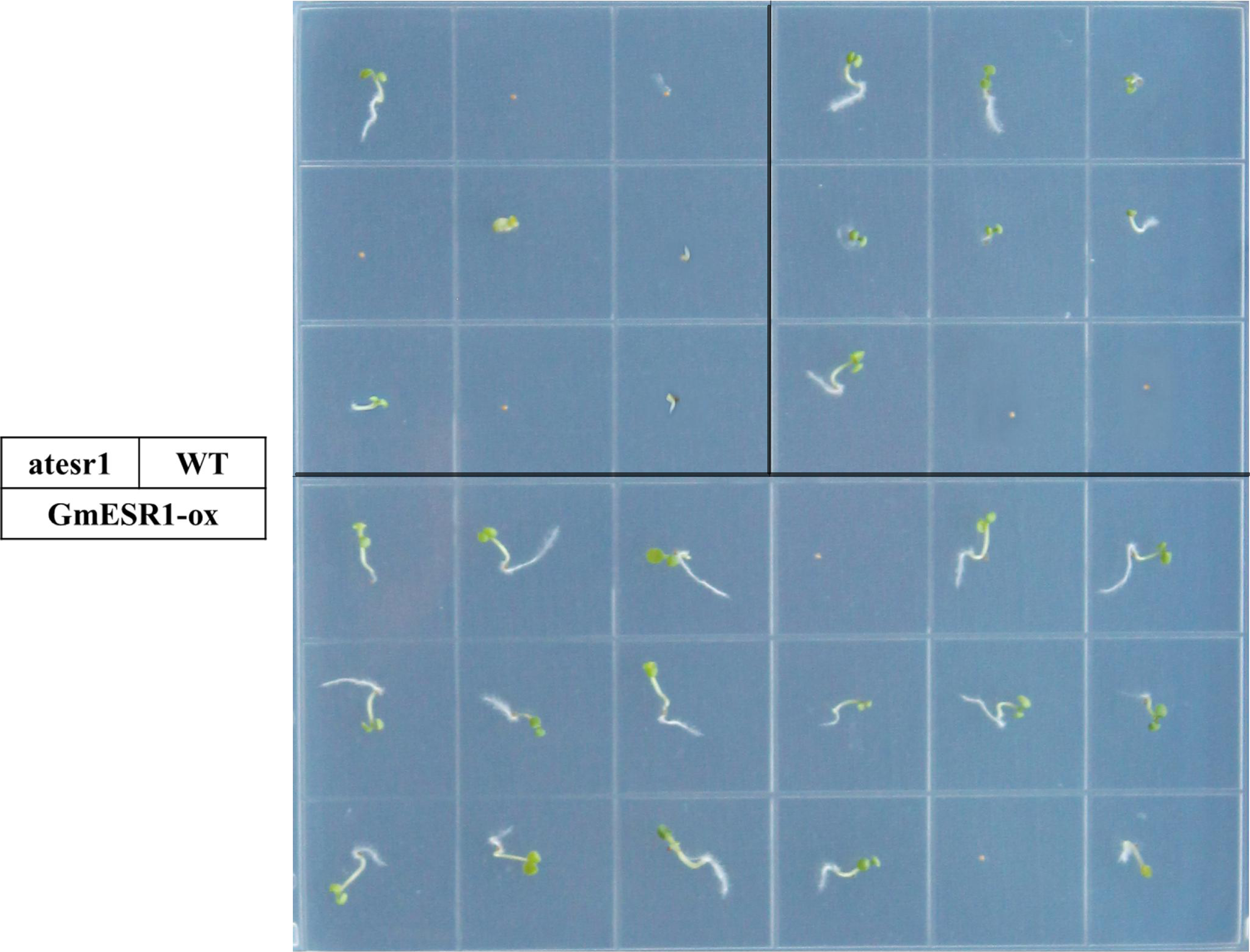

Supplement: S4 Fig — Two independent GmESR1-ox lines are included. (TIF) [file pone.0175656.s004.tif]

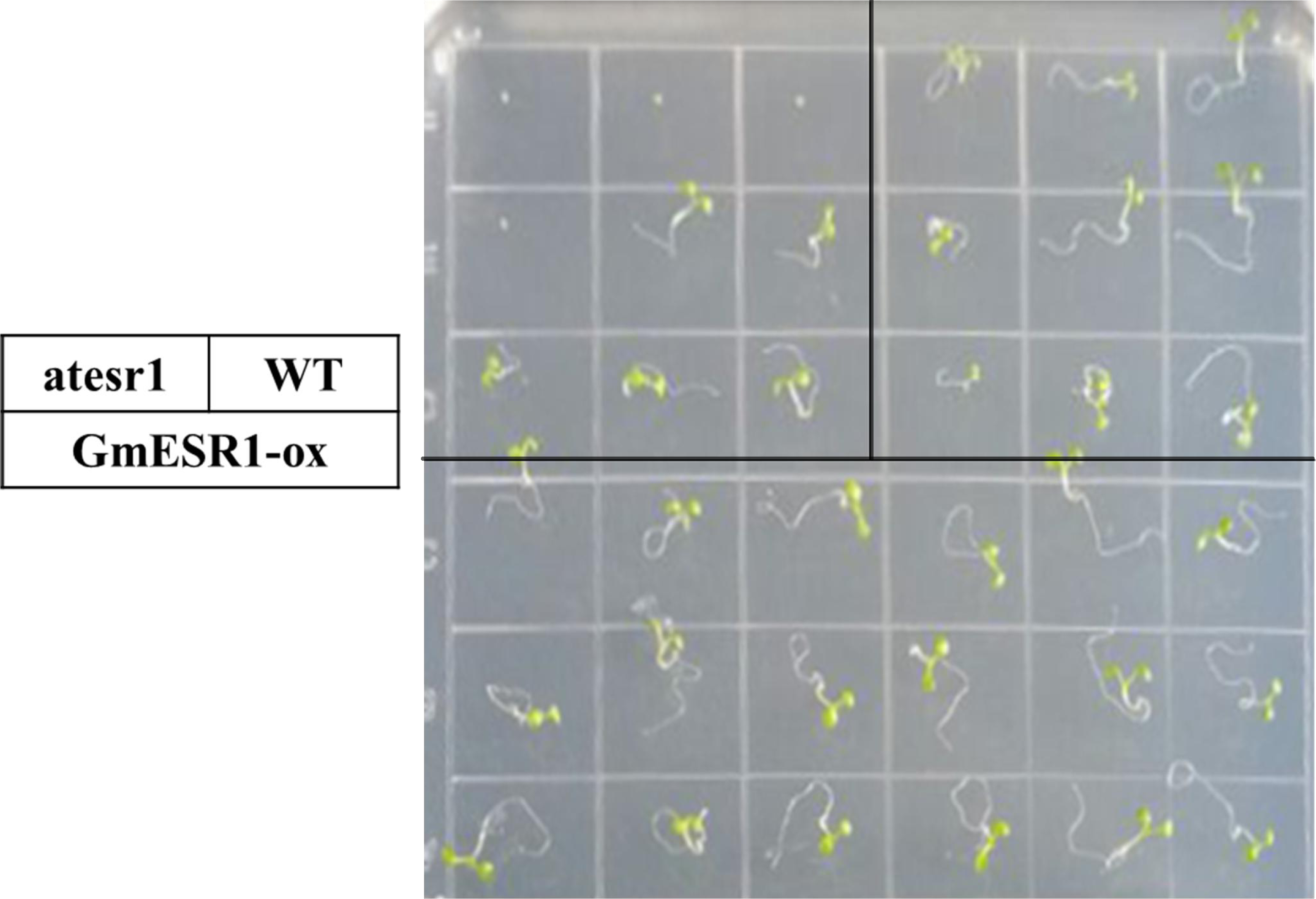

Supplement: S5 Fig — Two independent GmESR1-ox lines are included. (TIF) [file pone.0175656.s005.tif]
