# Supplementary material for: Functional analysis of the GmESR1 gene associated with soybean regeneration
Source: PLoS One. 2017 Apr 12;12(4):e0175656. doi: 10.1371/journal.pone.0175656 (PMC5389854; doi:10.1371/journal.pone.0175656)
Supplement: S1 Table — (DOC) [file pone.0175656.s006.doc]

S1 Table. Oligonucleotide primers used in this study.

|  | Name | Sequences |
| --- | --- | --- |
| Gene cloning | *GmESR1*-F | *BamH*I  5′ TTTGGATCCCTTCAGAAAGTATCACTAC 3′ |
|  | *GmESR1*-R | SacI  5′ TTAGAGCTCTAATTGAAAGGCCAGAC 3′ |
| qPCR | *GmESR1*-qF | 5′ CTCAGAACTTCCACGACCAG 3′ |
|  | *GmESR1*-qR | 5′ TTAACAGACAAAGAGCCTCCAC 3′ |
|  | *GmActin4*-F | 5′GTGTCAGCCATACTGTCCCCATTT3′ |
|  | *GmActin4*-R | 5′GTTTCAAGCTCTTGCTCGTAATCA3′ |
|  | *AtACTIN8*-F | 5′ CGTCCCTGCCCTTTGTACAC 3′ |
|  | *AtACTIN8*-R | 5′ CGAACACTTCACCGGATCATT 3′ |
| Screening of mutants | LP | 5′ TCGTGTTCCTCTCGTAAAACG 3′ |
|  | RP | 5′ GAGCACGAAAAGTTGGAGAAG 3′ |
|  | LB | 5′ ATTTTGCCGATTTCGGAAC 3′ |
| Over-expression | *bar*-F | 5′ GCGGTACCGGCAGGCTGAAG3′ |
|  | *bar*-R | 5′ CCGCAGGAACCGCAGGAGTG3′ |
| GmESR1 protein experiment | *GmESR1-*1F | *BamH*I 5′CGCGGATCCAATGAGGCGTCTCAACGGGGTAGCTC 3′ |
|  | *GmESR1-*1R | *Hind*III  5′CCCAAGCTTAGCATTTTGCATTCTGATTGCGAAA 3′ |
| sequence of probe | GCC-BOX  F | 5′ATCCATAAGAGCCGCCACTAAAATAAGACCGATCAA 3′ |
|  | GCC-BOX  R | 5′TTGATGGTCTTATTTTAGTGGCGGCTCTTATGGAT 3′ |
|  | mGCC-BOX  F | 5′ATCCATAAGATCCTCCACTAAAATAAGACCGATCAA3′ |
|  | m GCC-BOX  R | 5′TTGATCGGTCTTATTTTAGTGGAGGATCTTATGGAT 3′ |
